# Multiscale Modeling of Wobble to Watson–Crick-Like Guanine–Uracil Tautomerization Pathways in RNA

**DOI:** 10.3390/ijms22115411

**Published:** 2021-05-21

**Authors:** Shreya Chandorkar, Shampa Raghunathan, Tanashree Jaganade, U. Deva Priyakumar

**Affiliations:** Center for Computational Natural Sciences and Bioinformatics, International Institute of Information Technology, Hyderabad 500 032, India; shreya.chandorkar@research.iiit.ac.in (S.C.); shampa.santra@research.iiit.ac.in (S.R.); tanashree.jaganade@research.iiit.ac.in (T.J.)

**Keywords:** QM/MM, MD, Watson–Crick, WC-like, mispair, wobble, tautomerization, G•U, reaction energies

## Abstract

Energetically unfavorable Watson–Crick (WC)-like tautomeric forms of nucleobases are known to introduce spontaneous mutations, and contribute to replication, transcription, and translation errors. Recent NMR relaxation dispersion techniques were able to show that wobble (w) G•U mispair exists in equilibrium with the short-lived, low-population WC-like enolic tautomers. Presently, we have investigated the wG•U → WC-like enolic reaction pathway using various theoretical methods: quantum mechanics (QM), molecular dynamics (MD), and combined quantum mechanics/molecular mechanics (QM/MM). The previous studies on QM gas phase calculations were inconsistent with experimental data. We have also explored the environmental effects on the reaction energies by adding explicit water. While the QM-profile clearly becomes endoergic in the presence of water, the QM/MM-profile remains consistently endoergic in the presence and absence of water. Hence, by including microsolvation and QM/MM calculations, the experimental data can be explained. For the G•U${}^{enol}$→ G${}^{enol}$•U pathway, the latter appears to be energetically more favorable throughout all computational models. This study can be considered as a benchmark of various computational models of wG•U to WC-like tautomerization pathways with and without the environmental effects, and may contribute on further studies of other mispairs as well.

## 1. Introduction

The complementarity of purine and pyrimidine nucleobases (i.e., G-C and A-T) is essential to form the double stranded DNA helix as proposed by Watson–Crick [1]. However, occasional occurrence of the noncanonical mispairing of nucleobases (e.g., a base paired to one of its less likely tautomeric forms [1]) leads to spontaneous mutations. Nucleic acid bases due to the presence of their carbonyl and amino functional groups can exist in multiple tautomeric forms, i.e., keto–enol and amino–imino [1,2]. The “major” tautomers keto- and amino-forms are predominant under physiological conditions whereas, “minor” tautomers imino- and enol forms are rare. One such example is the guanine•uracil (G•U) mismatch, which typically forms the wobble structure with the two hydrogen bonds instead of three in a complementary WC G•C match.

**Figure 1:**
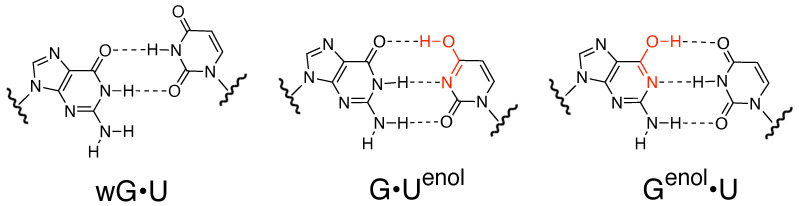
Structures of wobble G•U, and G•U${}^{enol}$, G${}^{enol}$•U WC-like mispairs.

RNA is a negatively charged [3,4] polymeric molecule essential in various biological roles, like, coding, decoding, regulation, and expression of genes. The RNA biochemistry is known to have notably influenced by tautomeric equilibria associated with guanine [5], e.g., recognition of ligands (xanthine/oxythiamine pyrophosphate) by purine riboswitch [6] and thiamine pyrophosphate riboswitch [7]. Studies of tautomeric equilibria faced numerous challenges due to the low abundance of the “minor” tautomers, fast exchange rates, and high chemical and structural similarities with their “major” counterpart [8]. However, in the quest of understanding the mechanism of tautomerism involving conformations/structures known as ’excited states’ (ESs) in biomolecules [9], various types of experimental and theoretical studies have been performed. Traditional spectroscopic techniques, such as, variable temperature NMR, 2-D IR [8,10], and X-ray crystallography [11,12,13,14,15,16] have been employed to characterize such tautomers. Theoretical studies by means of ab initio molecular orbital (MO), density functional theory (DFT) calculations [17,18,19,20], and molecular dynamics (MD) simulations [21,22,23] have been carried out in order to elucidate the transition mechanism. Recently, NMR relaxation dispersion techniques that enable the characterization of low-abundant, short-lived conformational states were used to study base tautomerization in DNA and RNA duplexes [24,25,26] by Al-Hashimi and co-workers. They demonstrated that wobble G•T/U mispairs exist in dynamic equilibrium with WC-like tautomeric (Figure 1) and anionic ESs, which can cause miscorporations during replication and translation processes.

Earlier computational studies [27] showed that rG•rU${}^{enol}$ is energetically more favorable compared to dG•dT${}^{enol}$ while considering their wobble to WC-like enolic transition pathways. This nicely corroborated to change the rapid rG•rU${}^{enol}$⇌ rG${}^{enol}$•rU equilibrium in favor of rG•rU${}^{enol}$ (40%) in RNA compared to dG•dT${}^{enol}$ (20%) in DNA. Previous studies have shown that tautomerization of the G•T mispair depends significantly on the environment. For instance, G•T mispair adopt wobble configuration in case of A- and B-DNA [11,28] whereas, depending on the polymerase variants G•T mispair adopts WC-like structure in the closed state [14,29] and wobble structure in the open state [30]. Further, it has been reported that local microenvironment can modulate the wobble G•T ⇌ WC-like enolic G•T equilibrium [31]. Several computational studies have revealed that different solvent environments can influence the thermodynamics and kinetics of the G•T tautomerization [18,32]. Recently, classical molecular dynamics (MD) simulations and the free energy perturbation data indicated that in the case of human DNA polymerase λ, among various G•T mispairs, G${}^{enol}$•T is predominant over G•T${}^{enol}$ and wobble structure [21]. Computational studies based on quantum mechanics/molecular mechanics (QM/MM) umbrella sampling simulations focused on the impact of environmental effects on G•T/U wobble to tautomerization mechanisms [33,34]. Free energy calculations on various systems, such as, isolated G•T mispair in aqueous, and the G•T base pair incorporated in A-DNA, B-DNA and DNA polymerase exhibited different stabilities for wobble G•T, G${}^{enol}$•T, and G•T${}^{enol}$ structures.

It is known that there is a high prevalence of non-canonical base pairs in RNA and of all studies on WC-mismatches, the mechanism of G•U ⇌ WC-like G•U enolic equilibrium is considerably less explored. However, implications of such mispair and its WC-like tautomeric forms were found to play crucial roles in nucleic acid catalysis [35,36], RNA-ligand recognition [6,8], expanding the decoding capacity of transfer RNA [37,38], and therapeutic applications [39]. Hence, in the present work, we have focused our investigation on the G•U mispair and its tautomeric variants. To this end, we have employed QM, MD, and QM/MM methods in order to characterize various intermediates and transition states. We have performed a systematic study in order to find the mechanistic pathway using appropriate theoretical methods as given below: (1) Ab initio calculation of the isolated base pair in gas phase. These results indicate that wG•U to WC-like tautomerization is exoergic in nature. (2) MD simulations in order to sample the phase space of the hp-GU-20 RNA and hp-GU-24 RNA in explicit water. As a result, a microsolvation environment surrounding the base pair was determined for further investigation. (3) Ab initio calculation of the base pair model within a microsolvent environment (considering only one water molecule). The obtained mechanistic pathway manifests an endoergic reaction. (4) QM/MM calculations incorporating ribosomal environment in explicit water surrounding the base pair. The resulting pathway shows similarities with that of the gas phase QM calculations considering the base pair with microsolvation. This indicates that modeling of such base pair transition using theoretical methods needs systematic benchmarking. QM/MM results specifically unveil that intrinsic effects originating from the immediate surrounding of the base pair might be the rationale of the experimentally observed populations of various G•U mispairs.

## 2. Computational Methods and Details

### 2.1. QM

Geometry optimizations of all base pairs including intermediates and transition states (TSs) along the wobble G•U ⇌ WC-like G•U pathway were carried out using the density functional theory (DFT) employing the Gaussian 16 [40] program package. The Becke3–Lee–Yang–Parr (B3LYP) [41,42,43,44,45] hybrid functional in combination with 6-311++G(d,p) [46,47,48,49] basis sets was used. We modeled the gas phase reaction first by taking only the base pair and then by including microsolvation along with the base pair. Intrinsic reaction coordinate (IRC) calculations in both directions following the normal mode of the imaginary frequency corresponding to a TS lead to a reactant and product.

### 2.2. MD

Initial structures and sequences for hp-GU-20 and hp-GU-24 RNA hairpins (untautomerised forms) were selected as the reference from the NMR experimental data of Al-Hashimi et al. [24,50]. The structures were further built using the VDfold3 server [51]. The sequence for the hp-GU-20 RNA loop is (5${}^{\prime }$-GCAGUGGCUUCGGCCGCUGC-3${}^{\prime }$) and, similarly for the hp-GU-24, the sequence used was (5${}^{\prime }$-GGCAGGUAGCUUCGGCUGCCUGCC-3${}^{\prime }$). We have performed MD simulations using the NAMD [52] program employing the CHARMM36 force field [53] and modified TIP3P water [54]. A pre-equilibrated waterbox of volume (64 Å)${}^{3}$ was used to solvate the RNA systems. Nineteen Na${}^{+}$ ions for hp-GU-20 and twenty-three Na${}^{+}$ ions for hp-GU-24 were also added to the system. Overlapping water molecules (defined to be within 2.4 Å of the RNA system) were removed. The model systems were solvated in this equilibrated solvent box by a 20 ps minimization followed by a 1 ns equilibration in NVT ensemble. Particle mesh Ewald (PME) was used to calculate the long range electrostatic interactions using a grid spacing of 1.0 Å. A cutoff of 12 Å was used for the short range Coulombic and Lennard–Jones interactions. The nonbonded interactions were truncated by using a switching function between 10 Å and 12 Å. During equilibration the backbone and side chain atoms of the RNA loop were restrained. The atom pair-lists were updated periodically at every 10 steps with an integration time-step of 2 fs. All bonds that include a hydrogen atom have been treated as rigid. In all the simulations, the terminal base pairs were harmonically constrained using force constants of 5 kcal/mol/Å${}^{2}$ to avoid base fraying throughout simulations. Periodic boundary conditions were used to simulate a continuous system. The trajectories of the molecules were calculated using the velocity Verlet algorithm. The SHAKE algorithm was used to constrain the length of covalent bonds involving hydrogen atoms [55]. The system was further subjected to a 100 ns of unconstrained production in NPT ensemble at a constant pressure of 1 atm using a Nosé–Hoover Langevin piston [56,57], and at 300 K temperature.

### 2.3. QM/MM

**Figure 2:**
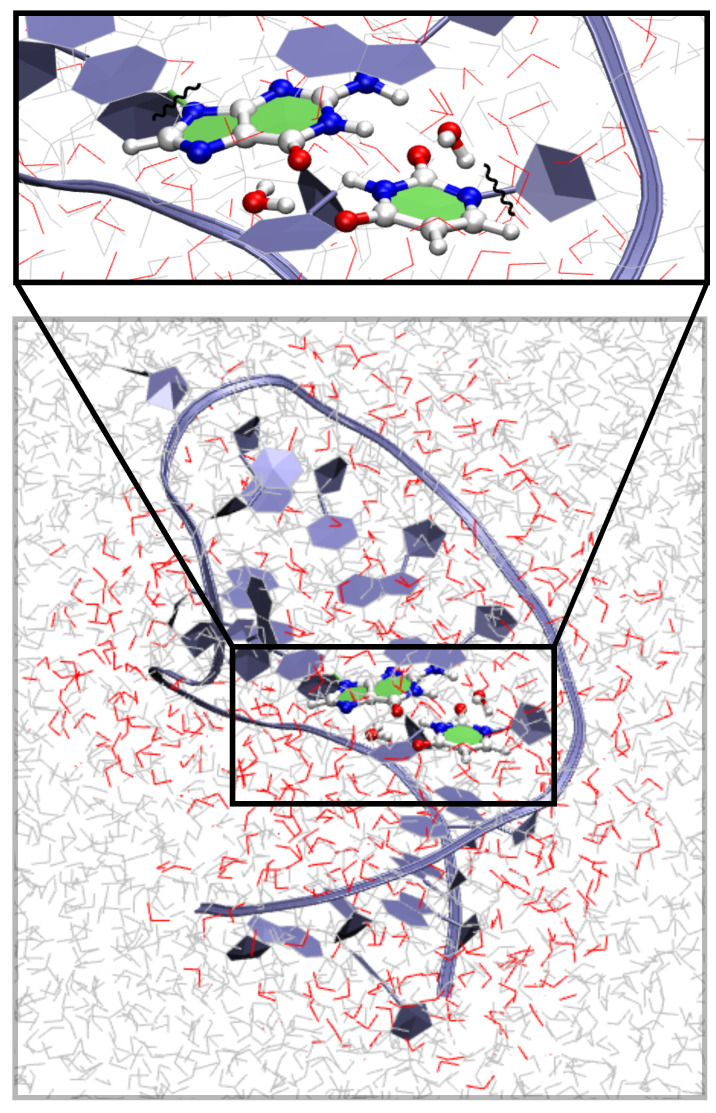
QM/MM simulation system. The G•U base pair (without the phosphate and sugar groups) and two water molecules (in balls and sticks representation) are included in the QM-region. Waters shown in red belong to the MM-active region, and the rest (gray) is the MM-frozen part.

We have prepared an initial structure for performing QM/MM calculations using a structure taken from the MD trajectory of hp-GU-20. Afterwards, we have replaced the atomic coordinates of the QM-region by optimized geometries of the reaction intermediates obtained from QM gas phase calculations. Structural alignments were done using the VMD [58] program. All of these initial structures were further optimized using QM/MM methods. QM/MM calculations were performed using the ChemShell [59] program interfacing the Orca [60] QM and CHARMM [61] MM program packages, making use of the electrostatic embedding with the link atom approach and charge shift scheme [62]. B3LYP/6-31G* level of theory (unless stated otherwise) with the combination of CHARMM36 [63] RNA force fields and TIP3P waters [64] were employed for all QM/MM calculations. For geometry optimizations, the DL-POLY [65] implementation with the DL-FIND optimizer [66] (for minimas) and HDLC optimizer [67] (for transition states) was used. Minima were located using the L-BFGS algorithm [68]. Transition states (TS) were found by a microiterative procedure that uses the P-RFO algorithm [69] for the reaction core employing an explicit numerical Hessian for eigenvector following, while the rest of the system is dealt with the L-BFGS scheme. All the optimizations were performed using HDLC coordinates [67]. The default convergence criteria [67] were used unless stated otherwise. Additionally, in case of TS, verification was done, so that, the available Hessian contained one negative eigenvalue corresponding to an appropriate transition vector. The QM part includes the G•U base pair (see Figure 2) consisting of 28 QM atoms including the two link atoms. The QM/MM model includes two waters, and therefore, has 34 QM atoms. The active region that is allowed to move during the optimization includes 3 base pairs above and below the G•U base pair and surrounding water molecules till 6 Å, beyond which the MM region was kept frozen. For a detailed illustration of various regions, see Figure 2. Infinite cut off was used for nonbonding MM and QM/MM electrostatic interactions. A QM/MM calculation for a particular structure, for example wG•U took about six times more computational time in comparison to a lone QM calculation, see Table S1 of the Supporting Information.

## 3. Results and Discussion

Reaction energies calculated using various theoretical approaches (QM and QM/MM) for the transition from wG•U to WC-like tautomers are presented and discussed in the following sections. Geometrical parameters (especially along the interface of the G•U mispair) of reaction intermediates and transition states are given for QM-pathways, and results obtained from analyses of MD trajectories from two different RNAs are shown as well.

### 3.1. QM Profile without Microsolvation

**Figure 3:**
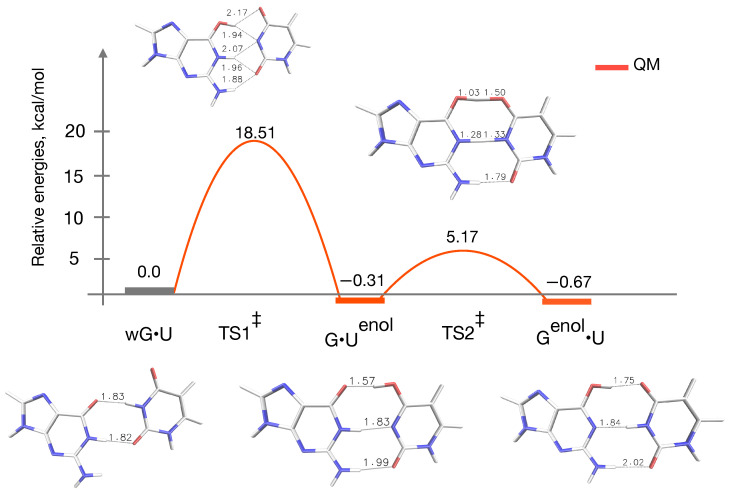
Reaction energy profile and geometries of reaction intermediates and transition states of the wG•U to WC-like tautomerization pathway obtained using QM calculations. Relative energies (kcal/mol) are calculated with respect to the wG•U base pair.

The reaction energy profile for the tautomerization of wG•U to G•U${}^{enol}$, followed by a double proton transfer reaction between G•U${}^{enol}$ and G${}^{enol}$•U is shown in Figure 3. The corresponding barrier energies are 18.5 and 5.2 kcal/mol, respectively, for TS1${}^{‡}$ and TS2${}^{‡}$. These results are quite similar to previous QM studies by Brovarets’ and Hovorun [20], and QM/MM MD studies by Pengfei et al. [33] in the case of wG•T to WC-like enolic G•T transitions. They have reported barriers of about ∼15–20 kcal/mol for TS1${}^{‡}$, and ∼4–7 kcal/mol for TS2${}^{‡}$. Figure 3 shows that G•U${}^{enol}$ and G${}^{enol}$•U have almost comparable energies with respect to the wG•U mispair. Besides, G${}^{enol}$•U is slightly more stable than the G•U${}^{enol}$, just by ∼0.4 kcal/mol. As a results, the G•U${}^{enol}$ → G${}^{enol}$•U transition is slightly energetically favorable.

This is quite similar to what has been found earlier in case of the G•T mispair [20,21,33]. The reaction free energies reported by Pengfei et al. indicates that wG•T → G•T${}^{enol}$ tautomerization is endoergic in aqueous solution, and in A-form and B-form DNA duplexes, but slightly exoergic in the DNA polymerase [33]. Unlike those, our present results show that wG•U → G•U${}^{enol}$tautomerization is slightly exoergic. However, previous studies using NMR relaxation dispersion technique measured 99% population of the ground state wobble G•T/U tautomers [24] indicating severe mismatch between the theoretical gas phase and experimental data. Nevertheless, at this point our understanding remains unclear whether obvious differences are arising because of the ribosomal G•U mispair, or aqueous environment or even both.

### 3.2. Conformational Space Sampling Using MD Simulations

**Figure 4:**
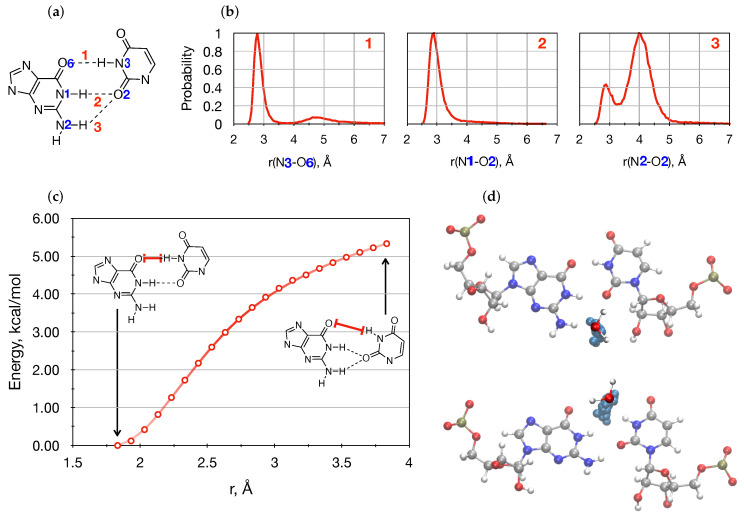
Analyses of MD trajectories reveal two distinct states: (**a**) Three donor–acceptor (DA) distances: **1** N3···O6, **2** N1···O2, and **3** N2···O2. (**b**) Probability distributions of three DA distances. (**c**) Relative energies of G•U base pair along reaction coordinate O···H corresponding to DA **1** computed at B3LYP/6-311++G(d,p). (**d**) G•U base pair is represented with balls and sticks, and averaged positions of water molecules are shown in Cyan colored balls in hp-GU-20 RNA.

To gain further insights into the ambiguous QM results of the gas phase G•U mispair, we have extended our computations using MD simulations for two different RNA systems, namely hp-GU-20 and hp-GU-24 RNA. Figure 4a depicts three different donor–acceptor (DA) distances: **1** N3···O6, **2** N1···O2, and **3** N2···O2. Figure 4b shows probability distributions of three DA distances along the trajectory of the hp-GU-20 RNA. Similar plots of the other hp-GU-24 RNA studied here are shown in the Supporting Information, Figure S1. These distributions clearly show the existence of three majorly populated peaks at the DA distance of about 2.8, 4.7, and 4.0 Å. It can be seen that DA **1** and **2** are almost 100% populated at about 2.8 Å. Additionally, the DA **1** arises at about 4.7 Å with below 10% population. Whereas, the DA **3** shows two peaks: One at about 2.9 Å, and the second at 4.0 Å with about 40% and 100% probabilities, respectively. It is evident that DA **1** and DA **3** are associated with two distinct populated states. Whereas, population of the DA **2** shows a single peak.

To gain further insights into the observed bifurcated populations of both DA **1** and **3**, we performed single point QM energy computations of the G•U base pair along the O···H coordinate associated with the DA **1**. Relative energies are plotted in Figure 4c. Interestingly, in the PES along the O···H reaction coordinate, two distinct structures were present [70]. One state seems to be energetically less favorable compared to the other state. However, from the equilibrium MD trajectories we did find that two states co-exist; interactions with water molecules paved a way for that. In fact, these two DA distances **1** and **3** are separated by waters. To this end, we found averaged-positions of two water molecules at two different states, see Figure 4d, top and bottom.

### 3.3. QM Profile Including Microsolvation

**Figure 5:**
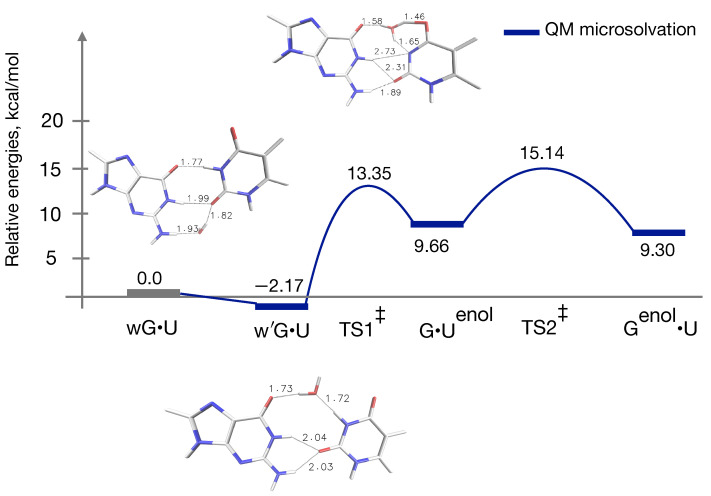
Reaction energy profile, and geometries of two types of wobble structures (wG•U and w’G•U) and one transition state $\mathrm{TS}1^{‡}$ along the wG•U to WC-like tautomerization pathway obtained using QM calculations with microsolvation, see Figure 3 for the remaining three structures G•U${}^{enol}$, $\mathrm{TS}2^{‡}$, and G${}^{enol}$•U, which are identical to those obtained from QM gas phase calculations. Here, one water molecule is present in addition to the G•U base pair. Relative energies (kcal/mol) are calculated with respect to the wG•U base pair.

Having found that interactions between the G•U base pair and water molecules play a significant role in modulating the stabilities of the two distinct states (wG•U and w’G•U), we have incorporated one water molecule along with the G•U base pair and performed geometry optimizations and frequency calculations for all intermediates and transition states (for energetics, see Figure 5).

Surprisingly, the relative energy of the $TS1^{‡}$ is lowered to 13.3 kcal/mol. In addition to that, the w’G•U structure, which turned out to be the most stable intermediate along the reaction energy profile, was located. This w’G•U structure has a relative energy of about −2.0 kcal/mol. Various wobble mismatches were reported earlier in order to predict novel pathways to connect WC-like G${}^{enol}$•T and G•T DNA base mispairs [20]; however, no water was included into the system. Interestingly, now wG•U → G•U${}^{enol}$ becomes endoergic as was reported in case of wG•T → G•T${}^{enol}$ tautomerization [33]. As a result of that, the relative energy of the TS2${}^{‡}$ increases to 15 kcal/mol, yet the G•U${}^{enol}$ → G${}^{enol}$•U transition is slightly favorable as was found earlier from QM reaction energy profile without the microsolvation. A point to be noted: In case of the QM-modeling of the G•U${}^{enol}$ → G${}^{enol}$•U transition, as the water molecule is not directly taking part, while calculating the relative energies of G•U${}^{enol}$ and G${}^{enol}$•U with respect to the wG•U structure, the energy of one water molecule calculated at the same QM-level of the theoretical methods (i.e., B3LYP/6-311++G(d,p)) was added with the respective intermediates and/or TS. The TS2${}^{‡}$ shows higher activation barrier with water compared to that of without the water (see Figure 5). However, the overall effects of the inclusion of the microsolvent environment in the QM-gas phase model cannot be overlooked.

The ambiguity over structural and energetical characterizations of the wobble to WC-like transformation with the incorporation of the water still lacks clear evidence because the TS1${}^{‡}$ without the water has five intermolecular H-bonding interactions (Figure 3). In the case of the gas phase QM calculations, the inclusion of the water seems to overcome a barrier that has the characteristic of an open configuration, see Figure 5. The desolvation of the (inter-base) hydrogen-bonding partners induces a favorable structural rearrangement. Besides, a slight non-planarity of the base pair is revealed. Earlier investigation predicted that the wobble to WC-like transition occurs without opening the base pair, and consequently tautomerizes to the WC-like base pair [20]. Until now, various studies addressed the wobble to WC-like transition in case of the G•U mispair considering the environmental contributions. However, conclusions drawn from them differ from exoergic and/or endoergic in closed-model and/or open-model [16,34,71,72,73,74]. Energies obtained from microsolvation seem to agree with experimental results [24] (ground state wG•U population >99%). This may indicate that solvation is important to get the qualitative trend correct. In order to further validate this we have performed QM/MM calculations on the hp-GU-20 RNA and obtained results are discussed in the following section.

### 3.4. QM/MM Profile

**Figure 6:**
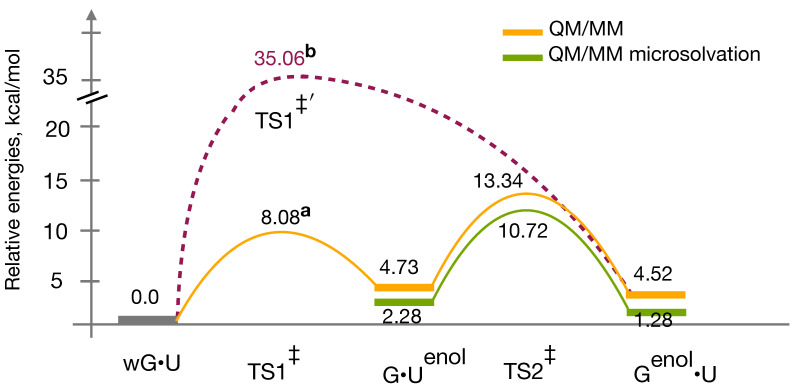
Reaction energy profiles of wG•U to WC-like tautomerization pathways obtained using QM/MM calculations. In case of the QM/MM energy profile with microsolvation two water molecules are included in addition to the G•U base pair in the QM-region. The energy profile depicted in purple colored dashed line is for wG•U → G${}^{enol}$•U pathway without the microsolvation. ${}^{a}$ and ${}^{b}$ refer to TS structures, which are obtained with slightly less tight convergence criteria, given in Tables S3 and S4 in the Supporting Information. Relative energies (kcal/mol) are calculated with respect to the wG•U base pair.

Studying chemical processes in DNA and RNA using the QM/MM approach which is known to describe the environmental effects of a system better compared to a smaller QM model, have become very popular [75,76,77]. In the current study, the computed reaction energy profile from the QM/MM calculations are presented in Figure 6. Here, again the relative energies of all intermediates and TSs are calculated with respect to the wobble G•U model. Firstly, wG•U → G•U${}^{enol}$ tautomerization process is consistently endoergic without and with inclusion of the microsolvation environment in the QM region. In the absence of water in the QM region, the relative energies of the WC-like G•U${}^{enol}$ and G${}^{enol}$•U structures were in the range of ∼5.0 kcal/mol, while in microsolvation environment their relative energies reduced to ∼2.0 kcal/mol. Consequently, Boltzmann population ratios of the G•U${}^{enol}$:G${}^{enol}$•U changed from 41:59 in absence of water to 16:84 in presence of water. The lone QM/MM values give an excellent agreement with the experimentally estimated ratios [24] however, microsolvated QM/MM values differ. Secondly, the G•U${}^{enol}$ → G${}^{enol}$•U transition is again slightly more favorable in both the cases. Figure S3 of the Supporting Information shows the geometrical parameters (specially along the interface of the G•U mispair) of reaction intermediates and transition states obtained from QM/MM calculations.

Understanding the exact role of solvents either in G•U/T ⇌ WC-like G•U${}^{enol}$/T${}^{enol}$ or in G•U${}^{enol}$/T${}^{enol}$⇌ G${}^{enol}$•U /T transitions is not yet clear as we have discussed in the preceding Section for QM models. From our QM/MM results, we see that water molecules kind of influence the microenvironment of the G•U base pair. It can be seen from the Figure S3c of the Supporting Information, the presence of water molecules tried to maximize their interactions with the base pair while keeping the planarity due the intra-base pair hydrogen bonding interactions. However, in case of the wG•U geometry, water molecules tried to orient themselves best to increase their interactions but not as efficiently as in enol forms. Therefore, stabilization of the enol forms is energetically more favorable compared to the wobble form. As a results, relative energies of the enol forms are reduced by about 2 kcal/mol at the microsolvated QM/MM level compared to the QM/MM alone.

One of the main objectives in this study was to calculate reaction barriers inclusively with the environmental effects coming from the solvent and the RNA by its very nature. However, locating TSs are not very straightforward specially, when various degrees of freedom are not only coupled intramolecularly but also intermolecularly. In particular, TS1${}^{‡}$, which has five intermolecular H-bonds including the bifurcated ones, was one of the most challenging to find out. In order to identify a TS, probably the most simplistic and intuitive approach is the constrained TS search. Here, a few (one and/or two) of the reaction coordinates along which the reaction may occur is chosen to be constrained while all other degrees of freedom are allowed to relax during the optimization. We have chosen two reaction coordinates for each constrained TS search. In the case of the TS1${}^{‡}$, two such 2-dimensional (2-D) constrained TS searches are depicted in Figure S2a,b in the Supporting Information. The highest energy point on the path can be considered as an approximation to the first-order saddle point–the true TS. The presence of a negative eigenvalue in the calculated Hessian matrix corresponding to the structure at the highest energy point in the potential energy surface (PES) ensures a possible TS of interest. Likewise, we were successful in locating a highest energy point (highlighted in Figure S2a in the Supporting Information). However, despite obtaining the negative eigenvalue, the displacement vectors for the normal mode did not correspond to TS1${}^{‡}$. Hence we did not consider it as a probable TS1${}^{‡}$. Imaginary frequencies of all valid transition states found in this study are listed in Table S2 of the Supporting Information.

Further, we have directly incorporated the TS1${}^{‡}$ geometry obtained from the QM gas phase calculation into the QM-subsystem of the QM/MM-setup. The frequency calculation of this particular structure gave us an imaginary frequency, and visualization of this normal mode indicated the correct atomic displacements related to the TS1${}^{‡}$. During the TS optimization with the default convergence criteria, the negative eigenvalue in the Hessian disappears, possibly due to the smaller magnitude of the eigenvalue. Therefore, we have reported one probable TS1${}^{‡}$ structure and its energy, which lies at about 8.0 kcal/mol in the PES.

In searching for a TS1${}^{‡}$ structure, another 2-D constrained TS search was performed, simply based on a chemical intuition, see Figure S2b in the Supporting Information. Here, we did find a TS that connects between the wG•U and G${}^{enol}$•U structures, with slightly less tight convergence criteria (for details see the Supporting Information). It lies quite high in the energy profile, by about 35 kcal/mol. In this transition state TS1${}^{‡\prime }$, formation of an N–H bond taking place concurrently with an N-H cleavage within this moiety G:N1···H···N3:U. Maybe this is an alternate pathway with a higher energy barrier (due to the non-planarity of the base pair) in comparison to the wG•U → G•U${}^{enol}$ transition. A similar non-planar TS was reported earlier, connecting a variant of the wobble geometries (authors named it as w2) and the G${}^{enol}$•T with a barrier as high as ∼40 kcal/mol. Brovarets’ and Hovorun predicted that, as the G•T${}^{enol}$→ G${}^{enol}$•T transition is energetically more favorable, the short lived G•T${}^{enol}$ structure transforms fast into the G${}^{enol}$•T mismatch, hence playing the crucial role indirectly in DNA replication [32]. Similarly, in the case of the G•U to WC-like transition also, G${}^{enol}$•U is most likely to play a significant role, albeit indirectly, because, the TS1${}^{‡\prime }$ (Figure 6) has a higher barrier compared to that of the TS1${}^{‡}$. Therefore, the wG•U will overcome the low-barrier TS1${}^{‡}$ and reach G•U${}^{enol}$, and would be followed by an exoergic G•U${}^{enol}$ → G${}^{enol}$•U transition via TS2‡, in spite of the marginal favorable stability of the G${}^{enol}$•U tautomer.

Very recently, Li et al. calculated QM/MM free energies using umbrella sampling simulations for G•T → WC-like G•T${}^{enol}$ transition in DNA, that is 15.7 kcal/mol, which is very close to the experimental free energy value of ∼16 kcal/mol [33]. Here, we are reporting QM/MM reaction energies for the G•U → WC-like G•U${}^{enol}$ transition in RNA, with a barrier of 8.08 kcal/mol (QM/MM without the microsolvation). Apart from the dissimilar base pairs, discrepancy may arise from various aspects, and could be explained as follows: (1) Even though both studies use QM/MM methods, results may vary due the computational protocols used therein, not only in terms of the exchange-correlation (XC) functionals/basis sets used but also the way free energies/reaction energies are calculated. Li et al. calculated the free energy barrier for the tautomerization process from the configurations space, which was approximately sampled using 13 reaction coordinates using the umbrella sampling techniques. In contrast, we have computed the barrier (static) from the fully relaxed geometries. Further, varying experimental thermodynamic conditions mentioned in [24] from the theoretical computations can also be one of the reasons. Nevertheless, locating the TS1${}^{‡}$ (QM/MM) was really challenging in this study. We need further investigation into QM/MM, dedicated solely to considering various XC-functionals, and sizes of the QM-region, number of water molecules included in the QM-region. Certainly, we cannot discard the possibility of variants of TS1${}^{‡}$ with slightly varying energy barriers.

**Figure 7:**
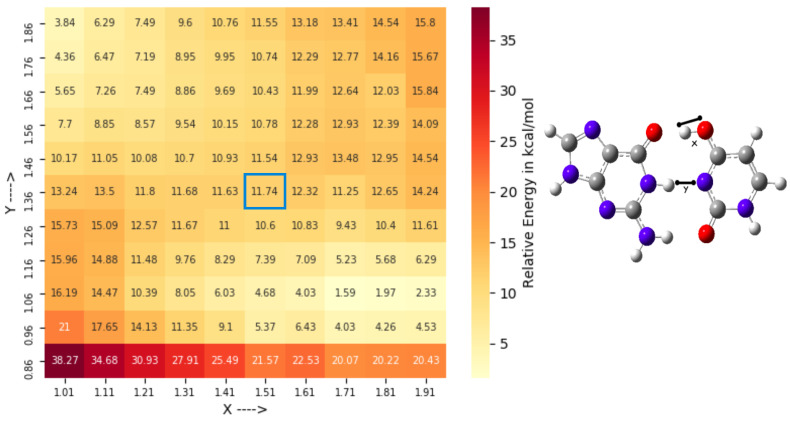
Reaction profile of the constrained TS search along the reaction coordinate *X* and *Y* highlighted in the balls and sticks representation of the G•U base pair for locating TS2${}^{‡}$ (6-31+G* basis sets used). Here, the QM-region consists of the G•U base pair along with two water molecules. The highlighted point was used for the TS optimization. Relative energies (kcal/mol) are calculated with respect to the wG•U base pair.

On the contrary, finding a TS2${}^{‡}$ structure was more straightforward. Figure 7 shows the energy profile of the corresponding 2-D constrained TS search (see Section S2.1 in the Supporting Information for details). The resulting TS connects between G•U${}^{enol}$ and G${}^{enol}$•U structures. The TS2${}^{‡}$ exhibits a double proton transfer. Similar TS structure was found earlier by computational studies connecting the WC-like G•T${}^{enol}$ and G${}^{enol}$•T tautomers [20].

## 4. Conclusions

We have modeled the wG•U → WC-like tautomerization pathways using various computational approaches, such as, QM, MD, and QM/MM. We have addressed the microenvironmental effects on the transition pathways as well. The low abundance, transient nature, and involvement of dynamic protons make characterizing these energetically unfavorable tautomeric bases a challenge. The methods that we have used could help to further study other WC-like mispair reactions, which otherwise have proved elusive to study experimentally. This could further help in understanding of nucleic acid catalysis, RNA–ligand recognition, and in the therapeutic mechanisms of nucleic acid base analogues. Furthermore, these methods help in studying their role in miscorporations during replication and translation, and could also help in drug design to prevent spontaneous mutations. All of our studied models revealed a slightly favorable stability of the G${}^{enol}$•U tautomer. The QM model with a single water molecule predicts an endoergic wobble to WC-like tutomerization, which was an exoergic process in the absence of water. Relative energies obtained from our QM model with microsolvation and QM/MM models agree with the experimentally obtained population percentages of the wobble and enolic structures; energies obtained from the gas phase QM results did not agree, because the enolic structures came out to be more favorable in comparison to wobble.

This clearly indicates that gas phase QM models have significant environmental effects on the reaction energies, corroborating a very recent study on the G•T mispair using QM/MM umbrella sampling free energy simulations [33]. However, QM/MM models offer an endoergic tautomerization process irrespective of the microenvironment. Within the microsolvation environment, the QM/MM reaction barrier is further lowered. What are the intrinsic interaction properties of ribosomal nucleobases, and what are the explicit waters that play significant yet characteristic roles in spontaneous mutations are still outstanding questions. This study sets a benchmark of various computational models employed to study the wobble G•U to WC-like tautomerization pathways with and without the environmental effects, and may contribute to further understanding the mispair tautomerization pathways related to other biological important mispairs as well.

## Data Availability

Data is contained within the article and supplementary material.

## Supplementary Materials

The following are available online at https://www.mdpi.com/article/10.3390/ijms22115411/s1, Figure S1: Probability distributions of donor-acceptor (DA) distances, Figure S2: Reaction profile of the constrained TS search for locating TS1${}^{‡}$ and TS1${}^{‡\prime }$, Figure S3: Geometries of intermediates and transition states computed using QM/MM methods, Table S1: Computation time for optimization of wG•U state in different methods with B3LYP/6-31G* level of theory, Table S2: Imaginary frequencies for transition states obtained using various computational methods, Tables S3 and S4: Convergence criteria for TS1${}^{‡}$ and TS1${}^{‡\prime }$, respectively.

## Abbreviations

The following abbreviations are used in this manuscript:

QM/MM	Quantum Mechanics/Molecular Mechanics
MD	Molecular Dynamics
DFT	Density Functional Theory
NMR	Nuclear Magnetic Resonance
WC	Watson–Crick
guanine•uracil	G•U
DNA	Deoxyribonucleic Acid
RNA	Ribonucleic Acid
