# Supplementary material for: Multiscale Modeling of Wobble to Watson–Crick-Like Guanine–Uracil Tautomerization Pathways in RNA
Source: Int J Mol Sci. 2021 May 21;22(11):5411. doi: 10.3390/ijms22115411 (PMC8196565; doi:10.3390/ijms22115411)
Supplement: Supplementary file 1 [file ijms-22-05411-s001.zip › ijms-1186632-supplementary.pdf]

Supporting Information: Multiscale Modeling of Wobble to Watson-Crick-like Guanine-Uracil Tautomerization Pathways in RNA

Shreya Chandorkar, Shampa Raghunathan, Tanashree Jaganade and U. Deva Priyakumar

Center for Computational Natural Sciences and Bioinformatics, International Institute of Information Technology, Hyderabad 500 032, India

1 Analyses of MD trajectories for the hp-GU-24 RNA

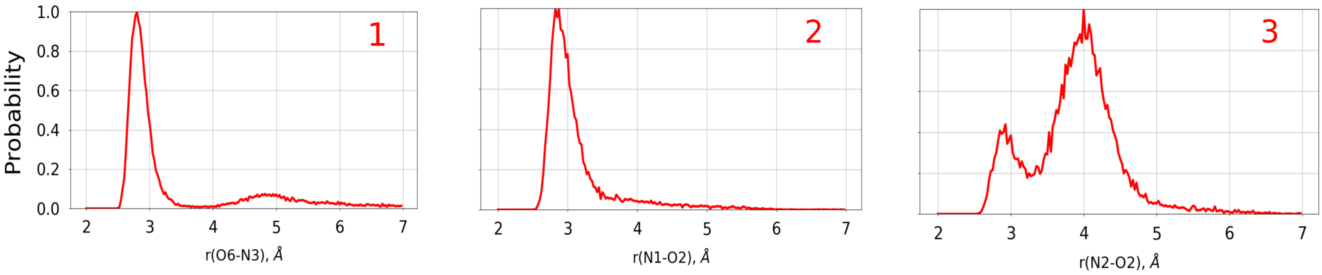

Figure S1: Probability distributions of donor-acceptor (DA) distances: **1** N3· · ·O6, **2** N1· · ·O2, and **3** N2· · ·O2 in case of the hp-GU-24 RNA system.

2 Constrained TS search using QM/MM methods

(a)

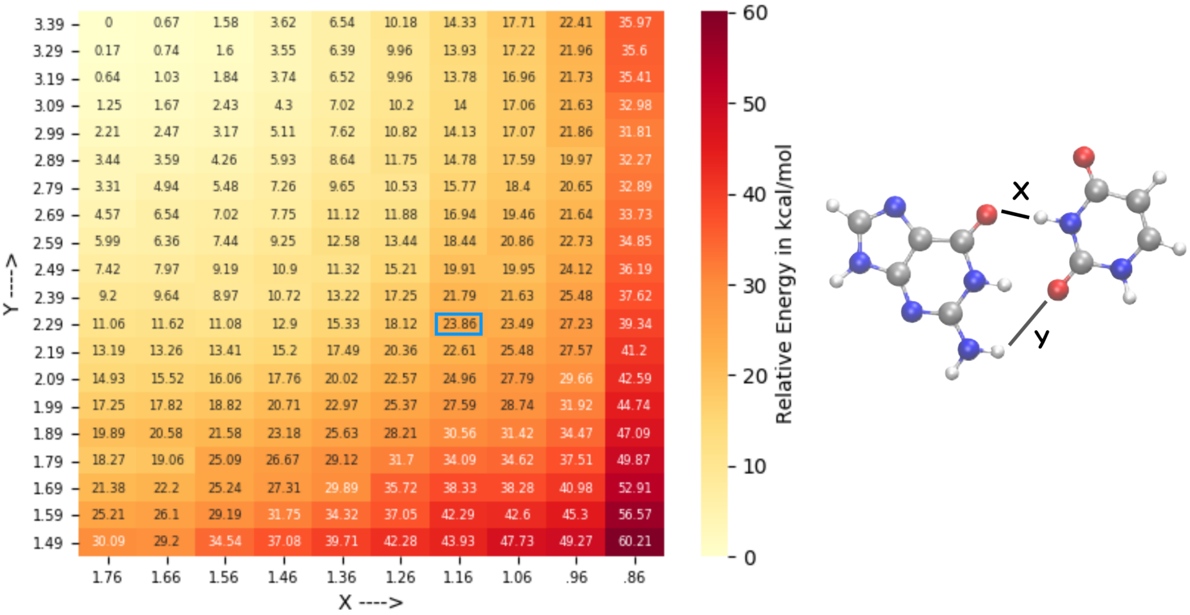

(b)

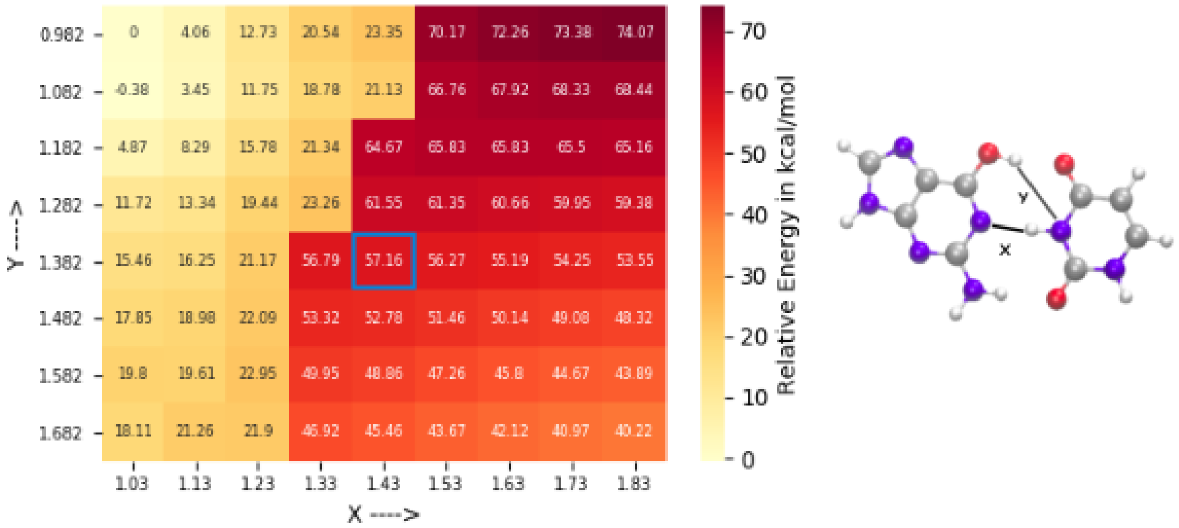

Figure S2: Reaction profile of the constrained TS search along the reaction coordinate X and Y highlighted in the balls and sticks representation of the G•U base pair for locating (a) TS1‡ and (b) TS1$^{\ddagger'}$. Relative energies (kcal/mol) are calculated with respect to the 1st structure: For (a) x=1.76 Å, y=3.39 Å, and for (b) x=1.03 Å, y=0.982 Å. In (b) along the x-axis there is large energy jump which might have arisen due to a significant structural change from x=1.23 to x=1.33.

2.1 Details of constrained TS2‡ search using QM/MM methods

The initial structure preparation for the potential energy scan was initiated by taking an MD snapshot. The same MD snapshot that was used to make wG•U was taken. Then, the starting structure for the 2-D scan was obtained by first replacing QM region coordinates with QM gas phase optimized G•U^{enol} structure and, was then given it for QM/MM optimization. Two water molecules were also included in the QM region along with G•U base pair in this scan. The highest energy point in the path was given for TS optimization which resulted in a TS with relative energy of 10.72 kcal/mol (QM/MM microsolvation, Figure 6 in the main text of the manuscript). This TS structure was again given for TS optimization

by considering only the G•U base pair in the QM region which resulted in a TS with relative energy of 13.34 kcal/mol (QM/MM, Figure 6 in the main text of the manuscript).

3 Intermediate structures and transition states from QM/MM calculations

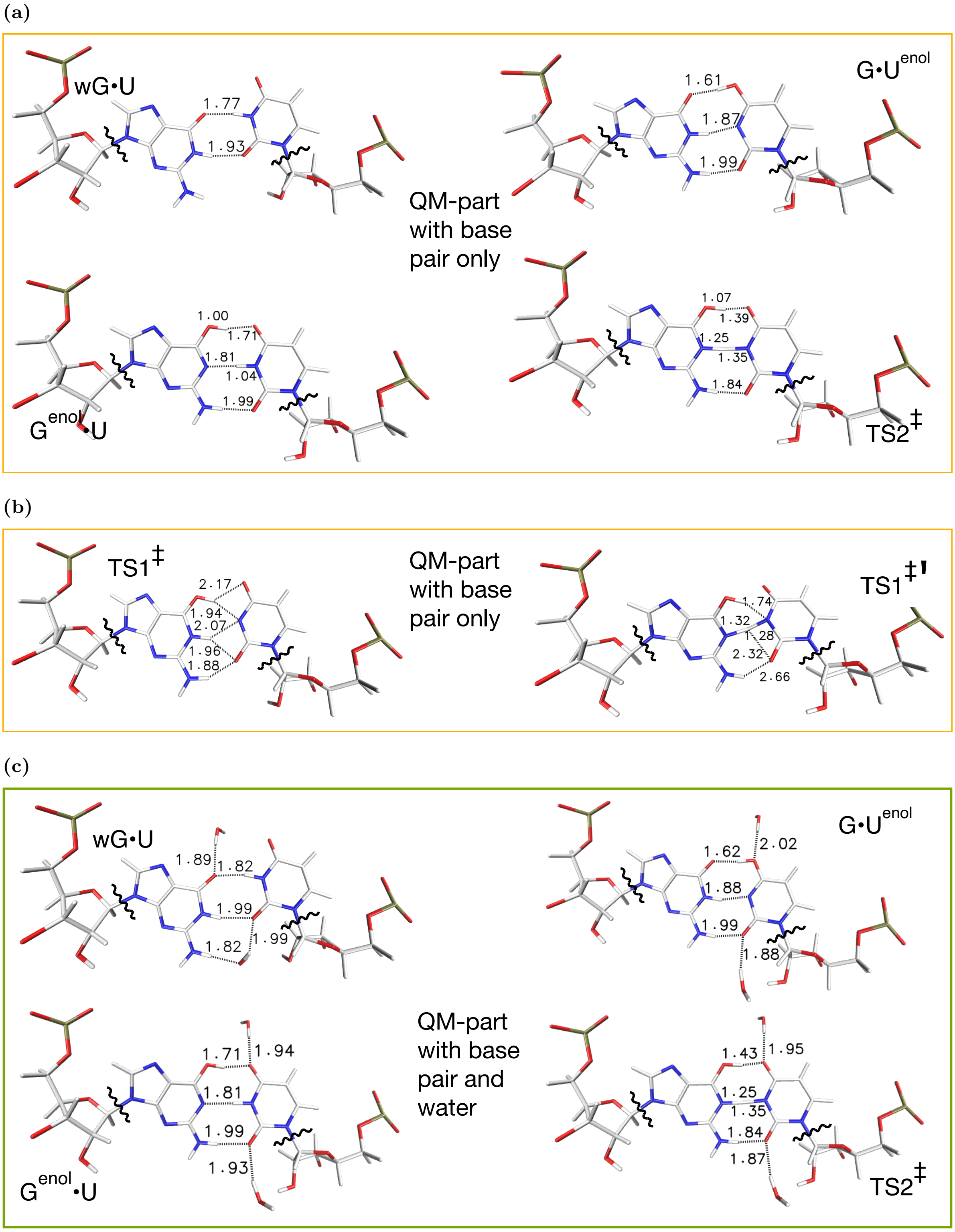

Figure S3: Geometries of intermediates and transition states along wG•U → WC-like tautomerization pathways computed using QM/MM methods where QM-part is calculated at B3LYP/6-31G* with different QM-regions: (a) and (b) base pair only; (c) base pair with two water molecules.

Table S1: Computation time for optimization of wG•U state in different methods with B3LYP/6-31G* level of theory

**Method**	**CPU Time**	**No. of QM atoms**
QM	1 hr	28
QM with microsolvation	1.5 hrs	31
QM/MM	6.2 hrs	28
QM/MM with microsolvation	6.8 hrs	34

Table S2: Imaginary frequencies for transition states obtained using various computational methods

**Method**	**Structure**	**Frequency (i cm^{-1})**
QM	TS1‡	161.2
	TS2‡	1180.5
QM with microsolvation	TS1‡	294.5
	TS2‡	1180.5
QM/MM	TS1‡	87.1
	TS2‡	1160.0
	TS1$^{\ddagger'}$	1292.1
QM/MM with microsolvation	TS2‡	1145.7

Table S3: Convergence criteria for TS1‡. All units are in a.u.

**Step/Grad**	**Actual**	**Target**
MaxStep	0.40022E-01	1.0000
RMSStep	0.25043E-02	1.0000
MaxGrad	0.26834E-02	0.45000E-03
RMSGrad	0.17567E-03	0.30000E-03

Table S4: Convergence criteria for TS1$^{\ddagger'}$. All units are in a.u.

**Step/Grad**	**Actual**	**Target**
MaxStep	0.32598E-02	0.54000E-01
RMSStep	0.18126E-03	0.36000E-01
MaxGrad	0.11572E-01	0.13500E-01
RMSGrad	0.33335E-03	0.90000E-02
